# *SPHK1*-*S1p* Signaling Drives Fibrocyte-Mediated Pulmonary Fibrosis: Mechanistic Insights and Therapeutic Potential

**DOI:** 10.3390/ph18060859

**Published:** 2025-06-09

**Authors:** Fei Lu, Gaoming Wang, Xiangzhe Yang, Jing Luo, Haitao Ma, Liangbin Pan, Yu Yao, Kai Xie

**Affiliations:** 1 Department of Medical Center, Soochow University, Suzhou 215000, China; lufei1112@alu.suda.edu.cn (F.L.); 20234256009@stu.suda.edu.cn (X.Y.); mhtszdx@163.com (H.M.); 2 Department of Thoracic Surgery, Xuzhou Central Hospital, Clinical School, Xuzhou Medical University, Xuzhou 221009, China; 100002008019@xzhmu.edu.cn; 3 Department of Medical School, Nanjing University, Nanjing 210000, China; 191230054@smail.nju.edu.cn; 4 Department of the First Clinical Medical College of Soochow University, Suzhou 215006, China; panliangbin1980@163.com; 5 Department of Respiratory Medicine, Nanjing Second Hospital, Nanjing University of Chinese Medicine, Nanjing 210002, China

**Keywords:** pulmonary fibrosis, *SPHK1*-*S1p* signaling, sphingolipid metabolism, therapeutic target, Mendelian Randomization

## Abstract

**Background:** Pulmonary fibrosis (PF) is a progressive interstitial lung disease characterized by chronic inflammation and excessive extracellular matrix deposition, with fibrocytes playing a pivotal role in fibrotic remodeling. This study aimed to identify upstream molecular mechanisms regulating fibrocyte recruitment and activation, focusing on the *SPHK1* pathway as a potential therapeutic target. **Methods:** We utilized Mendelian Randomization and phenome-wide association analyses on genes involved in sphingolipid metabolism to identify potential regulators of idiopathic pulmonary fibrosis (IPF). A bleomycin-induced mouse model was employed to examine the role of the *SPHK1-S1P* axis in fibrocyte recruitment, using SKI-349 to target *SPHK1* and FTY720 to antagonize *S1PR1*. **Results:** Our analyses revealed *SPHK1* as a significant genetic driver of IPF. Targeting *SPHK1* and S1PR1 led to a marked reduction in fibrocyte accumulation, collagen deposition, and histopathological fibrosis. Additionally, PAXX and RBKS were identified as downstream effectors of *SPHK1*. Our protein–protein interaction mapping indicated potential therapeutic synergies with existing anti-fibrotic drug targets. **Conclusions:** Our findings establish the *SPHK1-S1P-S1PR1* axis as a key regulator of fibrocyte-mediated pulmonary fibrosis and support *SPHK1* as a promising therapeutic target.

## 1. Introduction

Pulmonary fibrosis is a progressive and often lethal interstitial lung disease marked by excessive deposition of extracellular matrix, continuous activation of fibroblasts, and chronic inflammation, ultimately leading to irreversible scarring of the lung tissue [1,2]. The most severe and prevalent form is idiopathic pulmonary fibrosis (IPF), which is associated with a median survival rate of just 3 to 5 years. Currently available pharmacological treatments, such as Nintedanib and Pirfenidone, exhibit only limited effectiveness in slowing the progression of the disease [3,4]. While considerable advancements have been achieved in understanding its clinical features, the molecular mechanisms that underlie the condition remain only partially understood. This underscores the critical need for additional research to identify novel and effective therapeutic targets.

A hallmark of pulmonary fibrosis is the recruitment and activation of fibrocytes—circulating bone-marrow-derived mesenchymal progenitors with hematopoietic and stromal characteristics [5,6]. Following lung injury, fibrocytes migrate into the pulmonary microenvironment, where they differentiate into myofibroblasts. These cells play a crucial role in remodeling the extracellular matrix and produce important pro-fibrotic factors, such as transforming growth factor-beta (TGF-β), interleukin-13 (IL-13), and connective tissue growth factor (CTGF) [7]. Their accumulation correlates with disease severity, suggesting that fibrocytes are critical effectors in the pathogenesis of fibrosis. However, the precise molecular signals that regulate fibrocyte migration, differentiation, and activation in pulmonary fibrosis remain poorly defined.

Previous studies have highlighted the role of bioactive lipids, especially sphingolipids, in modulating immune cell trafficking and inflammation, positioning these molecules as key players in fibrotic diseases [8,9]. Sphingosine-1-phosphate (*S1P*) is a powerful signaling metabolite derived from sphingolipids, and it is synthesized by sphingosine kinase 1 (*SPHK1*). It regulates a wide range of biological functions through its interaction with five G-protein-coupled receptors (*S1PR1–S1PR5*). Among these, the *S1PR1* receptor has been implicated in cell migration, vascular barrier regulation, and immune cell recruitment [10,11]. Dysregulation of the *SPHK1*-*S1p*-*S1PR1* axis has been linked to pathological inflammation and tissue remodeling, but its role in fibrocyte-mediated pulmonary fibrosis remains unclear.

In this study, we sought to identify key therapeutic targets within the sphingolipid metabolism pathway that contribute to the pathogenesis of lung fibrosis and to elucidate how these targets, particularly *SPHK1*, influence the fibrotic process through their effects on fibrocytes. Initially, Mendelian Randomization (MR) analyses were conducted to explore causal associations between genes in the sphingolipid metabolism pathway and IPF, which led to the identification of *SPHK1* as a potential therapeutic target. Phenome-wide association study (PheWAS) analysis further suggested the safety and drugability of *SPHK1*. To validate these findings, we performed in vivo and in vitro experiments using a bleomycin (BLM)-induced mouse model of lung fibrosis. BLM treatment upregulated *SPHK1* expression, increased S1p levels, and elevated *S1PR1* expression in pulmonary mesenchymal cells, accompanied by a significant influx of fibrocytes into the fibrotic lung tissue. These results highlighted the role of *SPHK1*-*S1p*-*S1PR1* signaling in fibrocyte recruitment, activation, and differentiation. Moreover, the pharmacological inhibition of *SPHK1* or the functional antagonism of *S1PR1* disrupted fibrocyte trafficking, reduced extracellular matrix deposition, and ameliorated lung fibrosis, underscoring the therapeutic potential of targeting this pathway. To further investigate the systemic role of *SPHK1* in human IPF, we conducted mediation MR analyses, revealing plasma protein mediators of the *SPHK1*-driven pro-fibrotic response. Protein–protein interaction (PPI) analysis also uncovered functional overlap between *SPHK1* and established anti-fibrotic drug targets, suggesting that the modulation of *SPHK1*-*S1p* signaling could enhance the efficacy of current anti-fibrotic therapies.

## 2. Results

### 2.1. MR and PheWAS Support a Causal Role of SPHK1 in IPF

**Figure 1:**
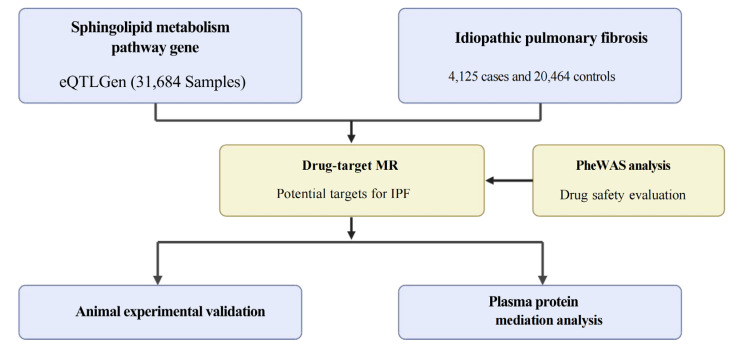
Flowchart. We conducted an integrative analysis that combined eQTL data from the eQTLGen consortium with IPF GWAS to identify potential pathogenic targets within the sphingolipid metabolism pathway. Causal genes were assessed using Mendelian Randomization, followed by a safety evaluation based on PheWAS. Top targets were further validated through animal experiments and plasma protein mediation analysis. MR: Mendelian Randomization; IPF: idiopathic pulmonary fibrosis; PheWAS: phenome-wide association study.

Figure 1 illustrates the flowchart representing the entire study.

**Figure 2:**
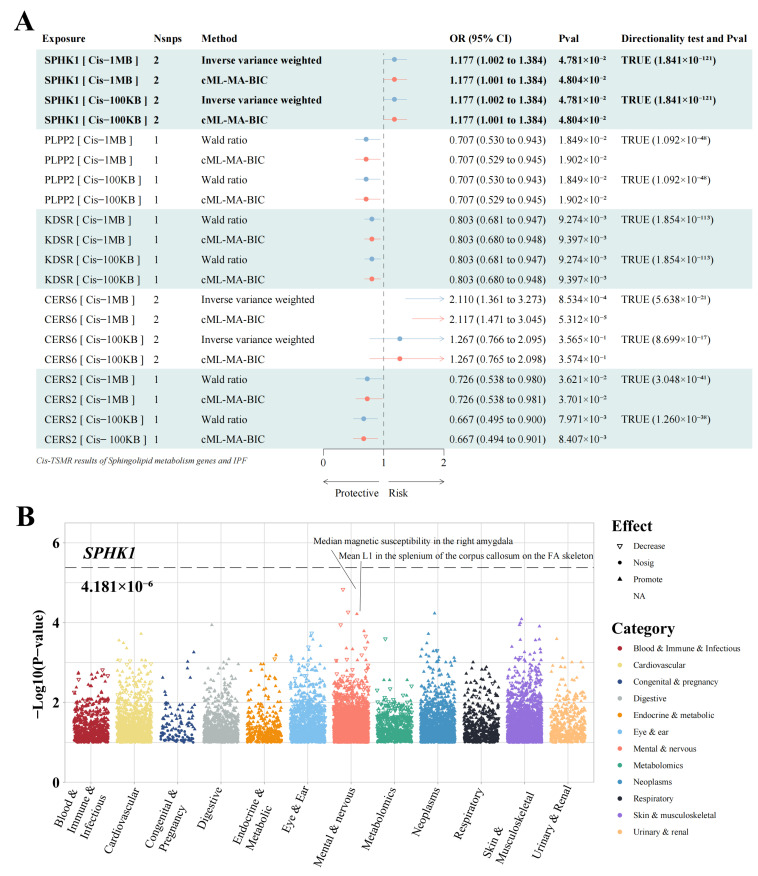
Identification of potential pathogenic targets of IPF using MR and PheWAS analyses. (**A**) TSMR results for sphingolipid metabolism pathway genes and IPF. (**B**) PheWAS results for *SPHK1*. TSMR: two-sample Mendelian Randomization; OR: odds ratio; CI: confidence interval; cML-MA: constrained maximum likelihood and model averaging; BIC: Bayesian information criterion; IPF: idiopathic pulmonary fibrosis.

To identify the potential pathogenic drivers of IPF within the sphingolipid metabolism pathway, we searched the GeneCard database for related genes and identified 106 targets (Supplementary Table S2). Gene eQTL data were extracted from the eQTLGen database, and MR analysis was conducted. The results from both the IVW and Wald ratio methods revealed five genes within a 1 MB cis-region that were causally associated with IPF. Specifically, the high expression of *CERS6* (OR: 2.110, 95%CI: 1.361–3.273, *p* = 8.534 × 10^−4^) and *SPHK1* (OR: 1.177, 95%CI: 1.002–1.384, *p* = 4.781 × 10^−2^) was positively correlated with an increased risk of IPF, while the elevated levels of PLPP2 (OR: 0.707, 95%CI: 0.530–0.943, *p* = 1.849 × 10^−2^), KDSR (OR: 0.803, 95%CI: 0.681–0.947, *p* = 9.274 × 10^−3^), and *CERS2* (OR: 0.726, 95%CI: 0.538–0.980, *p* = 3.621 × 10^−2^) were negatively associated with IPF risk (Figure 2A and Supplementary Table S3).

Importantly, these results maintained their consistency even after accounting for pleiotropy, showing no indications of reverse causality. Furthermore, most positive associations were evident under a stricter cis-definition (100 KB), except for *CERS6*. In conclusion, *SPHK1* appears to be less affected by pleiotropy, making it a more valuable pathogenic target with significant biological implications in IPF. These results indicate that *SPHK1* may be crucial in the pathogenesis of IPF by affecting sphingolipid metabolism.

To further evaluate the safety of *SPHK1* as a therapeutic target, we conducted PheWAS to assess its potential pleiotropic effects across 11,958 phenotypes spanning 12 categories. Under stringent statistical correction (*p* = 0.05/11,958), no significant associations were observed. The most notable traits—such as “Median magnetic susceptibility in the right amygdala (*p* = 1.475 × 10^−5^)” and “Mean L1 in the splenium of the corpus callosum on the FA skeleton (*p* = 5.473 × 10^−5^)”—were categorized under mental and nervous system phenotypes. These findings are consistent with the low pleiotropic potential observed in our MR analysis and suggest that *SPHK1* is a promising and safe target for therapeutic exploration (Figure 2B and Supplementary Tables S4 and S5).

While computational analyses strongly implicate *SPHK1* in IPF pathogenesis, experimental validation in relevant biological models is required to confirm its role in disease progression and elucidate underlying mechanisms. To achieve this, we employed a bleomycin (BLM)-induced mouse model of lung fibrosis to investigate the molecular function of *SPHK1* in pulmonary fibrosis.

### 2.2. Dysregulated Sphingolipid Metabolism in BLM-Induced Lung Fibrosis

**Figure 3:**
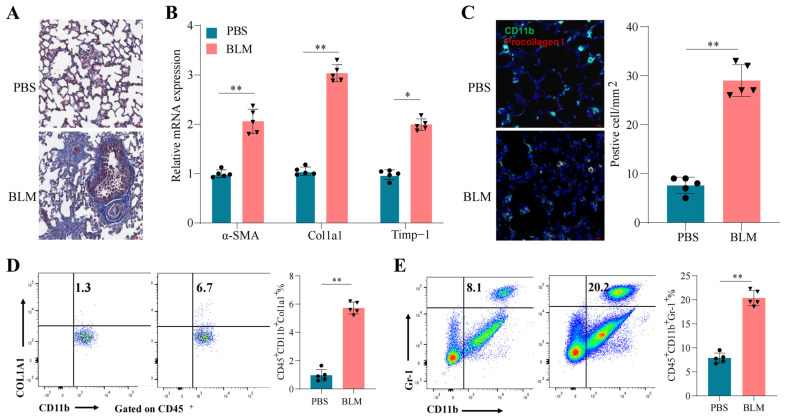
Bleomycin induces lung fibrosis, profibrotic gene upregulation, and immune-cell infiltration. (**A**) Masson’s trichrome staining. Scale bar: 50 µM. (**B**) mRNA levels of the indicated genes in lung tissues were determined using real-time PCR. (**C**) Lung sections from PBS- or bleomycin (BLM)-treated naive mice were assessed for CD11b (green) and collagen I (red) (**left**), along with the quantitation of immunostained cells in the lung (**right**). Scale bar: 50 µM. (**D**,**E**) Flow cytometry analysis of CD11b^+^collagen I^+^ fibrocytes (**D**) and CD11b^+^Ly6G^+^ neutrophils (**E**) in the lung. Statistical comparisons were performed using a two-tailed unpaired *t*-test. Error bars indicate the mean ± SD. * *p* < 0.05, ** *p* < 0.01. n = 5 independent biological samples.

Bleomycin is an established agent for inducing pulmonary fibrosis in animal models and has been shown to mimic features of acute diffuse interstitial pneumonia and chronic lung fibrosis in humans [1,12]. Consistent with previous reports, lung sections from BLM-treated mice in our study displayed extensive fibrosis characterized by Masson’s trichrome staining and collagen deposition (Figure 3A). Molecular analysis revealed the upregulation of key fibrosis markers, including Timp1, Col1a1, and α-SMA (Figure 3B). Additionally, immunohistochemistry and flow cytometry uncovered significant infiltration of CD11b^+^/collagen I^+^ fibrocytes and CD11b^+^Ly6G^+^Ly6C^+^ neutrophils in the lungs of BLM-treated mice, whereas these cell populations were minimal in control lungs (Figure 3C–E).

**Figure 4:**
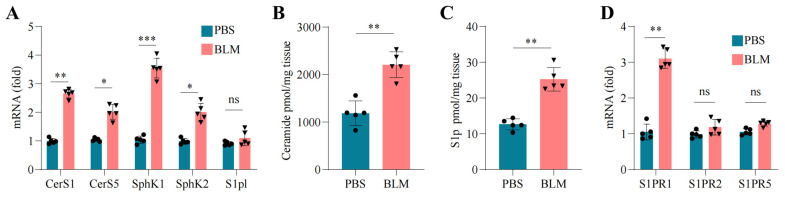
Dysregulated sphingolipid metabolism in BLM-induced lung fibrosis. (**A**) Real-time PCR assessment of mRNA levels for genes associated with the sphingolipid metabolism pathway in the lungs of mice treated with PBS or BLM. (**B**) Levels of ceramide in the lungs of PBS- or BLM-treated mice. (**C**) Levels of S1p in the lungs of PBS- or BLM-treated mice. (**D**) Real-time PCR analysis of S1p receptors in the mRNA of lungs from PBS- or BLM-treated mice. Statistical analyses were conducted using a two-tailed unpaired *t*-test. Error bars represent the mean ± SD. * *p* < 0.05, ** *p* < 0.01, *** *p* < 0.001, ns: non-significant. n = 5 independent biological samples.

Fibroblasts are critical for tissue remodeling and fibrosis, with their functional regulation likely involving various metabolic pathways, notably sphingolipid metabolism, which is emerging as a significant area of research. Sphingolipid molecules serve not only as vital components of cell membranes but also play active roles in signal transduction, impacting processes such as cell growth, differentiation, and apoptosis [13]. In the context of fibrosis, dysregulation of sphingolipid metabolism may influence the recruitment and differentiation of fibroblasts, potentially worsening disease progression. While these correlational findings are persuasive, there is a lack of in vivo evidence demonstrating that sphingolipid enzymes and receptors are dysregulated during lung fibrosis and that such dysregulation is associated with fibrocyte trafficking. To fill this gap, we analyzed key components of the pathway using the well-established BLM model of pulmonary fibrosis. Real-time PCR analysis confirmed the increased lung expression of sphingolipid metabolism pathway enzymes, including *SPHK1* and *SPHK2*, in BLM-treated mice. Conversely, sphingosine-1-phosphate lyase 1 (S1PL), which degrades S1p, remained unchanged (Figure 4A). This upregulation of *SPHK1* corresponded with the elevated levels of ceramide (Figure 4B) and S1p (Figure 4C), which is indicative of dysregulated sphingolipid metabolism. A further analysis of lung immune cells revealed a marked increase in the expression of S1p receptor 1 (*S1PR1*) (Figure 4D). This finding suggests that increased *SPHK1-S1p-S1PR1* signaling contributes to fibrocyte recruitment and trafficking to fibrotic lungs. The increased expression of *S1PR1* in fibrotic lungs underscores its potential involvement in fibrocyte migration in fibrotic environments.

### 2.3. SPHK1 Inhibition Attenuates Fibrocyte Accumulation and Lung Fibrosis

**Figure 5:**
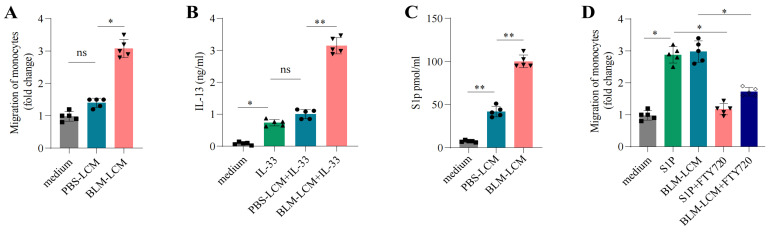
Lung-derived factors enhance fibrocyte chemoactivity and function. (**A**) In vitro migration of peripheral fibrocytes in response to conditioned medium (PBS-LCM or BLM-LCM) from the lungs of PBS- or BLM-treated mice. (**B**) Lung fibrocytes from PBS- or BLM-treated mice were cultured with IL-33, and the levels of IL-13 were measured in the medium. (**C**) S1p concentrations in the conditioned media from lung tissues of PBS- or BLM-treated mice. (**D**) S1PR mRNA levels in lung fibrocytes from PBS- or BLM-treated mice. Error bars represent the mean ± SD. * *p* < 0.05, ** *p* < 0.01, ns: non-significant.

To investigate whether S1p and its associated pathways contribute to fibrocyte recruitment, we cultured murine peripheral fibrocytes with lung-conditioned medium (LCM) from PBS- or BLM-treated mice. Chemotaxis assays showed that fibrocyte migration was significantly higher in response to BLM-LCM compared to PBS-LCM (Figure 5A). Additionally, fibrocytes cultured with BLM-LCM showed increased IL-13 production in the presence of IL-33 (Figure 5B). Consistent with the increased *SPHK1* expression, S1p levels were significantly elevated in BLM-LCM compared to PBS-LCM (Figure 5C). To further validate the role of *SPHK1*-*S1p* signaling, we employed FTY720, a functional antagonist of *S1PR1*, to assess its impact on fibrocyte migration [14,15]. Exposure to LCM or S1p significantly enhanced fibrocyte migration, whereas treatment with FTY720 effectively and markedly suppressed S1p- and LCM-mediated migration (Figure 5D). These findings indicate that *SPHK1*-*S1p* signaling may be essential in regulating the fate of fibrocytes.

**Figure 6:**
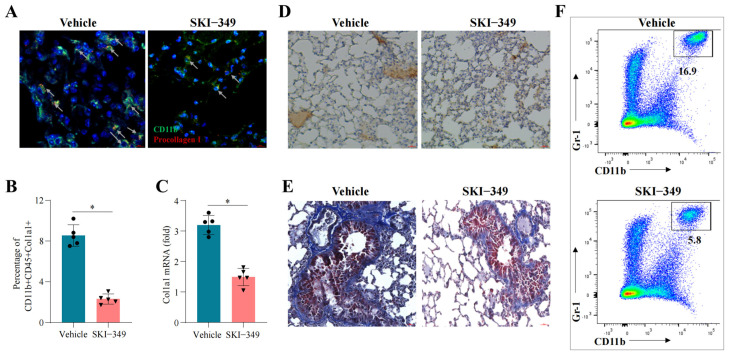
*SPHK1* inhibition attenuates fibrocyte accumulation and lung fibrosis. (**A**–**F**) C57BL/6J mice were treated with SKI-349 (10 mg/kg, intraperitoneally (i.p.)) every other day after BLM treatment. (**A**,**B**) Immunofluorescence (**A**) and flow cytometry (**B**) analyses of CD11b^+^collagen I^+^ fibrocytes in the lungs of mice that received a single injection of BLM and were subsequently treated with either PBS (vehicle) or SKI-349. CD11b (green) and collagen I (red). Scale bar: 50 µM. The arrow indicates the double-positive fibrocytes. (**C**) mRNA level of col1a1 in the lung. (**D**) IHC analysis of collagen I in the lung. (**E**) Masson’s trichrome staining. Scale bar: 50 µM. (**F**) Flow cytometry of CD11b^+^Ly6G^+^ neutrophils in the lung. Statistical comparisons were conducted using a two-tailed unpaired *t*-test or one-way ANOVA with Tukey’s multiple-comparison test. Error bars represent the mean ± SD. * *p* < 0.05. n = 5 independent biological samples.

To determine whether blocking *SPHK1*-derived S1p signaling could mitigate fibrocyte activity and reduce lung fibrosis, we used SKI-349, a selective *SPHK1* inhibitor, in the BLM model. Mice were administered BLM on day 0, followed by intraperitoneal injections of SKI-349 every other day for 14 days. SKI-349 treatment significantly decreased the number of fibrocytes in lung tissues, as evidenced by histological analysis and flow cytometry (Figure 6A,B). Furthermore, treatment markedly suppressed *Col1a1* mRNA and protein expression (Figure 6C,D) and reduced fibrosis severity when assessed using histopathological scoring (Figure 6E). Interestingly, SKI-349 therapy also decreased neutrophil infiltration (Figure 6F), underscoring the broader anti-inflammatory effects of *SPHK1* inhibition. These results align with our in vitro findings, where SKI-349 attenuated fibrocyte migration in response to S1p and lung-conditioned media, suggesting that *SPHK1* inhibition disrupts the pro-fibrotic microenvironment established in BLM-treated lungs.

### 2.4. Mediation Analysis Reveals Downstream Pathways and Therapeutic Implications

**Figure 7:**
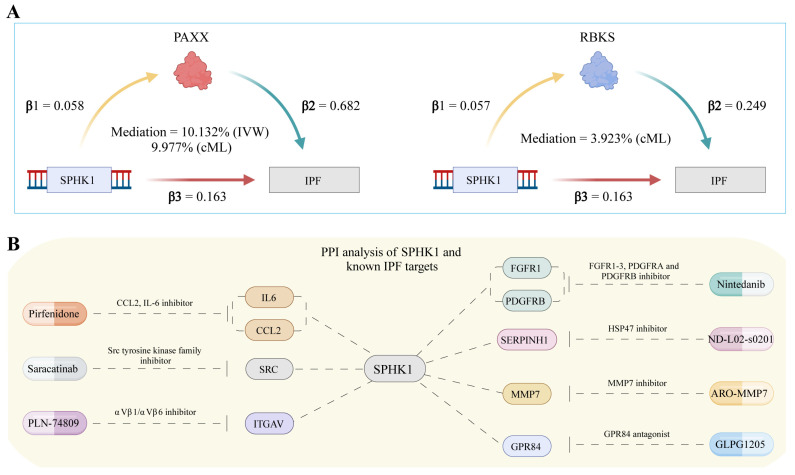
Mediation analysis and protein interaction networks. (**A**) Mediation MR results for SPHK1 showing that it mediates plasma protein to IPF; (**B**) PPI analysis network for *SPHK1* and known potential IPF targets. IVW: inverse variance weighted; IPF: idiopathic pulmonary fibrosis; PPI: protein–protein interaction.

To explore the mechanisms underlying *SPHK1*’s pathogenic role in IPF, we conducted a mediation analysis to identify plasma protein pathways influenced by *SPHK1*. A total of 379 positive causal pathways were identified, linking *SPHK1* to plasma protein alterations, and 1264 causal plasma protein–IPF pathways were detected. After excluding pathways with reverse causality or inconsistent directional associations, *PAXX* emerged as a key mediator with significant effects in both the IVW (mediation effect proportion = 10.132%) and cML-MA (mediation effect proportion = 9.977%) methods. *RBKS* also mediated *SPHK1*-associated IPF risk, with a mediation effect proportion of 3.923% after pleiotropy adjustment (Figure 7A and Supplementary Table S6). PCR analysis confirmed the associations between *SPHK1*, *PAXX*, and *RBKS* expression.

PPI network analysis suggested that *SPHK1* interacts with nine known IPF therapeutic targets, which involve seven existing drugs. Notably, *SPHK1* was found to interact with *FGFR1* and *PDGFRB*, targets of Nintedanib, as well as IL6 and CCL2, targets of Pirfenidone. These interactions suggest that the current IPF treatments may indirectly modulate *SPHK1*’s expression, thereby influencing its pathogenic role. These findings provide important insights into potential combination therapies targeting *SPHK1* alongside established treatments such as Nintedanib and Pirfenidone (Figure 7B).

## 3. Discussion

Pulmonary fibrosis is a progressive and frequently lethal condition marked by excessive deposition of extracellular matrix, abnormal activation of fibroblasts, and persistent inflammation. Pulmonary fibrosis is a progressive and frequently lethal condition marked by excessive deposition of extracellular matrix, abnormal activation of fibroblasts, and persistent inflammation [16]. Although the etiology of fibrosis varies across diseases, such as IPF and secondary fibrosis induced by environmental factors, the dysregulation of various cellular pathways contributes broadly to the fibrotic process [17]. In this study, we emphasize the crucial role of sphingolipid metabolism—specifically, the *SPHK1*-*S1p* signaling pathway—in promoting fibrosis by influencing fibrocyte recruitment, differentiation, and activation.

Our study indicates that *SPHK1*-*S1p*-*S1PR1* signaling is a critical regulator of fibrocyte-mediated pulmonary fibrosis. Fibrocytes, monocyte-derived mesenchymal progenitors, were markedly enriched in fibrotic lungs following BLM treatment and expressed both hematopoietic and stromal markers [18]. Consistent with their role as a major cellular source of extracellular matrix proteins and cytokines in fibrosis, these fibrocytes were highly activated in BLM-treated lungs. Fibrocyte accumulation was closely associated with increased *SPHK1* expression and *S1p* production in lung tissues, suggesting that the pro-fibrotic microenvironment created by *SPHK1* activation facilitated fibrocyte recruitment and migration. This finding aligns with previous studies that demonstrate that fibrocytes play a crucial role in remodeling injured tissue by orchestrating extracellular matrix deposition and secreting pro-fibrotic mediators, such as TGF-β and IL-13, which contribute to the persistence of fibrosis [19,20].

Our findings support previous studies indicating that dysregulation of sphingolipid metabolism contributes to the progression of fibrotic disease, including IPF [21,22]. Furthermore, the role of *S1PR1* in cell migration and survival underscores its importance in fibrocyte recruitment during fibrosis [23,24]. These observations highlight the significance of the *SPHK1-S1p-S1PR1* axis in remodeling fibrotic characteristics. The *SPHK1*-*S1p* signaling axis emerged as a key molecular pathway linking fibrocyte activity to fibrotic progression [25,26]. Previous studies have demonstrated that *SPHK1* expression is elevated in the lungs of IPF patients and BLM-treated mice. Additionally, *SPHK1* inhibition has been shown to alleviate pulmonary fibrosis and enhance survival in animal models [27,28,29]. These findings implicate *SPHK1* in the pathogenesis of fibrosis through TGF-β signaling and sphingolipid dysregulation. Expanding on this, our study uncovers a unique fibrocyte-centered mechanism through which the *SPHK1-S1P-S1PR1* axis promotes fibrosis, highlighting a previously underappreciated connection between sphingolipid metabolism and the dynamics of monocyte-derived progenitor cells. *S1p*, a bioactive sphingolipid metabolite, is well recognized for its regulatory roles in immune cell trafficking, vascular integrity, and cellular migration. Elevated S1p levels in fibrotic lungs suggest their involvement in the recruitment of fibrocytes and the activation of mesenchymal cell populations, as previously suggested in fibrosis and cancer models [30]. Our findings further highlight that the inhibition of *SPHK1*, the enzyme responsible for S1p biosynthesis, using SKI-349 significantly ameliorated fibrosis. Importantly, SKI-349 not only decreased fibrocyte accumulation but also inhibited collagen gene expression and the histological characteristics of fibrosis, highlighting its potential as a therapeutic target. The role of the *S1PR1* receptor in mediating S1P’s effects was further supported by using FTY720, a functional antagonist of *S1PR1* [31,32]. FTY720 abrogated fibrocyte trafficking to the lungs and alleviated inflammation, further emphasizing the importance of the *SPHK1*-*S1p*-*S1PR1* axis in fibrotic processes. Together, these findings highlight a feed-forward loop in which *SPHK1*-driven *S1p* bioavailability supports a fibrotic microenvironment conducive to fibrocyte recruitment, retention, and activation.

MR analysis reinforces the pathogenic role of *SPHK1* in IPF, supporting earlier reports that *SPHK1* mRNA and protein levels are significantly upregulated in fibrotic lung tissue and in TGF-β-stimulated fibroblasts [33]. The current MR data extend these findings by demonstrating a causal association between genetically determined *SPHK1* expression and the risk of IPF in humans. Mediation analysis further identified the plasma proteins *PAXX* and *RBKS* as statistically significant downstream effectors of *SPHK1*.

From a therapeutic perspective, PPI network analyses revealed physical and/or functional connections between *SPHK1* and several established IPF drug targets for IPF, including FGFR1 [34] and PDGFRB [35] (targets of Nintedanib), as well as IL-6 [36] and CCL2 [37] (targets modulated by pirfenidone). In vitro studies suggest that S1P transactivates FGFR and PDGFR signaling cascades [9]. These findings suggest that *SPHK1* inhibition could potentially enhance the effects of Nintedanib or Pirfenidone or that the clinical activity of those agents may, in part, arise from the indirect attenuation of sphingolipid signaling. This creates the intriguing possibility that *SPHK1* inhibition could synergize with existing therapies, enhancing their therapeutic efficacy. Moreover, these interactions suggest that Nintedanib and Pirfenidone may partially modulate sphingolipid signaling, which warrants further investigation.

A major hallmark of fibrosis is the dynamic interplay between immune and stromal cells in the lung microenvironment. Fibrocytes, as a bridge between hematopoietic and stromal compartments, are uniquely positioned to regulate this crosstalk. The persistent influx of fibrocytes in fibrotic lungs represents a critical point of intervention, as newly recruited fibrocytes are more responsive to S1p-mediated activation compared to resident fibrocytes. Strategies aimed at disrupting fibrocyte trafficking, such as *S1PR1* antagonism or chemokine receptor blockade (e.g., CXCR2 inhibition), could complement *SPHK1*-targeted therapies by addressing both fibrocyte migration and activation. By disrupting pathological fibrocyte dynamics, it may be possible to reprogram the fibrotic microenvironment and restore tissue homeostasis. While our study provides insights into the mechanisms by which *SPHK1*-*S1p* signaling regulates fibrocyte-mediated fibrosis, several questions remain. First, the relative contributions of *SPHK2*, another enzyme responsible for S1p production, deserve further exploration. Second, the role of non-fibrocyte-derived cell populations, such as macrophages and neutrophils, in modulating sphingolipid metabolism and fibrosis warrants additional investigation. Finally, the interplay between systemic metabolic alterations and local sphingolipid signaling in fibrotic diseases remains poorly understood.

In conclusion, this study identifies dysregulated sphingolipid metabolism, particularly *SPHK1*-*S1p* signaling, as a central mechanism driving fibrocyte recruitment and activation in pulmonary fibrosis. By elucidating the molecular and cellular underpinnings of fibrosis, we propose targeting *SPHK1*-*S1p* signaling as a promising strategy to mitigate fibrosis and reprogram pathological tissue remodeling. These findings pave the way for future research aimed at refining therapeutic approaches for fibrotic diseases and improving patient outcomes.

## 4. Materials and Methods

### 4.1. Data Sources

The genome-wide association studies (GWASs) and expression quantitative trait locus (eQTL) data utilized in this study are detailed in Supplementary Table S1. The GWAS data for IPF were sourced from the International IPF Genetics Consortium, encompassing large-scale meta-summary data from 4125 cases and 20,464 controls [38]. Gene eQTL data specific to the sphingolipid metabolism pathway were retrieved from the eQTLGen project, which includes 31,684 samples [39]. In addition, protein quantitative trait loci (pQTL) data from the UKBPPP, comprising 34,557 participants [40], were employed to analyze the potential downstream mechanisms related to core target plasma proteins. All study populations are of European descent, and the data are publicly available in accordance with the informed consent and regulations of the respective sources.

### 4.2. Mendelian Randomization

The criteria for selecting instrumental variables (IVs) for the target genes are as follows: (1) non-rare risk loci with a minor allele frequency (MAF) greater than 0.01; (2) loci that are significantly associated with gene expression, defined by a *p*-value of <5 × 10^−8^; (3) non-linkage disequilibrium loci located within the cis-regulatory region (1 MB upstream and downstream of the gene), with r^2^ < 0.001 and KB = 10,000; (4) robust IVs, indicated by an F-statistic of >10 [41]. We evaluated the causal association between sphingolipid metabolism genes and IPF using the TwoSampleMR package with default parameters while excluding palindromic sequences with MAF values between 0.42 and 0.58. When only one effective IV was available, we applied the Wald ratio method; for scenarios involving multiple IVs, the inverse variance weighted (IVW) method was employed to estimate the causal effect. A *p*-value of <0.05 was deemed indicative of a positive causal association. Given that Cochran’s Q test and the MR Egger intercept test are not suitable for assessing heterogeneity and pleiotropy when IVs are sparse, we utilized the random-effects IVW method along with constrained maximum likelihood and model averaging (cML-MA) to mitigate potential heterogeneity and pleiotropic bias [42]. The random-effects model reduces the overconcentration of causal estimates due to IV heterogeneity, while the cML-MA method adjusts for pleiotropic bias. To further minimize potential pleiotropy induced by cis-regulatory definitions at the gene level, we conducted a replication analysis using a 100 KB cis-region to assess the robustness of the results based on the 1 MB cis-definition. Additionally, we performed a directionality test to investigate potential reverse causality. We performed a two-step MR mediation analysis to evaluate the potential plasma protein mediation pathways through which the core targets contribute to the development of IPF. The underlying assumption of this approach is that the MR coefficients from exposure to mediator and mediator to outcome should align in direction with the exposure to outcome, and both should be statistically significant. We used bootstrap resampling with 1000 random samples to estimate the indirect effect, and the proportion of the mediation effect was calculated as the ratio of the Beta of the indirect effect to the Beta of the exposure–outcome effect. Mediation analysis was conducted for both IVW and cML-MA MR pathways to identify as many potential mediation pathways as possible.

### 4.3. PheWAS

To assess the potential pleiotropy of the target gene and its drug safety profile, we conducted a PheWAS using data from the UK Biobank cohort, which comprises over 500,000 participants from diverse ancestral backgrounds. To mitigate pleiotropy, the analyzed features were selected to exclude confounders associated with IPF [43,44].

### 4.4. Protein–Protein Interaction

To explore potential protein interactions between the core target and previously known IPF targets, we used the Genemania (https://genemania.org/, accessed on 19 February 2025) web tool to analyze the interaction patterns between *SPHK1* and targets of IPF treatment drugs reported in clinical trials. The potential association was visualized by constructing a network diagram.

### 4.5. Animal Model Establishment

Female C57BL/6 mice (specific pathogen-free (SPF) grade, 6 weeks old, weighing 16–18 g) were anesthetized with 1% sodium pentobarbital (60 mg/kg) and subjected to the intranasal instillation of bleomycin sulfate (Sigma B1141000, Sigma-Aldrich, St. Louis, MO, USA; 2 mg/kg body weight) to establish the experimental model. Mice received intranasal administration of either PBS control or bleomycin sulfate (BLM). Lungs were collected 14 days after bleomycin treatment. This study was approved by the Institutional Animal Care and Use Committee (IACUC) of the Experimental Animal Center at the Medical Center of Soochow University (the Fourth Affiliated Hospital of Soochow University, Suzhou Dushu Lake Hospital) (Approval No. 241191). The study was conducted in compliance with the ARRIVE guidelines [45].

### 4.6. Hematoxylin and Eosin (H&E) Staining

Tissue samples were fixed in 4% paraformaldehyde overnight at 4 °C, dehydrated using an ethanol gradient, cleared with xylene, and embedded in paraffin. Serial paraffin sections, each 4–5 μm thick, were prepared and mounted on glass slides. Sections were deparaffinized, rehydrated, and stained with hematoxylin for 5 min, followed by differentiation with acid alcohol. After rinsing with water, sections were counterstained with eosin for 2 min. Finally, the slides were dehydrated, cleared, and mounted. Histological alterations were observed and imaged using a light microscope. Images obtained from H&E staining were quantified using the software ImageJ2 version 2.3.0.

### 4.7. Masson’s Trichrome Staining

Paraffinized tissue sections were deparaffinized and rehydrated prior to staining. Collagen deposition was evaluated using a Masson’s trichrome staining kit (Solarbio, Beijing, China; G1340) following the manufacturer’s instructions. Briefly, sections were treated with Bouin’s solution at 56 °C for 1 h to enhance staining. Sections were then sequentially stained with Weigert’s iron hematoxylin, Biebrich scarlet-acid fuchsin, and phosphomolybdic acid, followed by aniline blue staining. After dehydration and clearing, sections were mounted with a resinous medium. Collagen fibers were visualized as blue, while muscle fibers and cytoplasm were stained red. Images were captured under a light microscope for further analysis.

### 4.8. Immunofluorescence (IF)

Tissue cryosections (6 μm thick) were prepared from frozen OCT-embedded samples. Sections were fixed in 4% paraformaldehyde (Solarbio, P1110) for 15 min at room temperature, then washed with DPBS (Gibco, Waltham, MA, USA; 14190250), and blocked for 1 h at room temperature using DPBS containing 5% bovine serum albumin (BSA, Sigma, A9418). Subsequently, sections were incubated overnight at 4 °C with primary antibodies, including anti-CD11b (1:200, Abcam, Cambridge, UK; ab133357) and rabbit anti-mouse polyclonal anti-collagen I (COL-I, 1:200, Abcam, ab34710). After washing, sections were incubated with Alexa Fluor 488-labeled goat anti-rat IgG (1:500, Invitrogen, Carlsbad, CA, USA; A11006) for anti-CD11b and biotin-conjugated goat anti-rabbit IgG (1:500, Abcam, ab6720), followed by Alexa Fluor 594-conjugated streptavidin (1:500, Invitrogen, S11227) for anti-COL-I. Nuclei were counterstained with DAPI (1 μg/mL, Thermo Fisher Scientific, Waltham, MA, USA; D1306) for 5 min, and sections were mounted with the Vectashield antifade mounting medium. Immunofluorescence images were captured using a confocal microscope. Fluorescence intensity was quantified using the software ImageJ. The mean fluorescent intensity (MFI) and percentage of positively stained regions were calculated across multiple fields of view.

### 4.9. Immunohistochemistry (IHC)

Paraffin-embedded tissue sections (4–5 μm thick) were deparaffinized in xylene and rehydrated through graded ethanol solutions. Antigen retrieval was performed by boiling the sections in sodium citrate buffer (10 mM sodium citrate, 0.05% Tween-20, pH 6.0, Solarbio, C1060) for 15 min under high pressure. Subsequently, sections were treated with 3% hydrogen peroxide (Solarbio, P1080) for 10 min at room temperature to block endogenous peroxidase activity. After washing with DPBS, slides were incubated with Protein Block Buffer (Vector Laboratories, Newark, CA, USA; X0909) for 30 min at room temperature to reduce nonspecific binding. Sections were then incubated overnight at 4 °C with a primary antibody against collagen I (1:100, Abcam, ab34710). After washing, sections were treated with HRP-conjugated goat anti-mouse/rabbit secondary antibody (1:200, GeneTech, South San Francisco, CA, USA; GP016129) for 30 min at room temperature. Immunoreactivity was visualized using a DAB substrate kit, with hematoxylin (Solarbio, G1120) being used as a counterstain. Afterward, slides were dehydrated, cleared, and mounted with a neutral resin. Images were acquired using a light microscope, and the staining area was quantified using the ImageJ software.

### 4.10. Fibrocyte Isolation from Peripheral Blood

Fibrocytes were harvested and cultured as previously described [46,47]. Briefly, total PBMCs first were isolated from murine blood through density gradient centrifugation (1000× *g* for 20 min) and cultured overnight on fibronectin-coated plates (6-well plates, 5 × 10^6^ PBMCs/well) in DMEM supplemented with 20% FCS. The nonadherent cells were then removed by a single, gentle aspiration. Following 7 days of continuous culture, the adherent cells were lifted by incubation in cold 0.05% EDTA/PBS and were depleted by immunomagnetic selection of contaminating T cells (anti-CD4/CD8 MicroBeads, Miltenyi Biotec, Gaithersburg, MD, USA), neutrophils (Anti-Ly-6CMicroBeads), and B cells (anti-CD19 MicroBeads). Fibrocyte purity was verified to be >95% by FACS analysis.

### 4.11. Chemotaxis Assay

The QCM™ Chemotaxis 96-Well Cell Migration Assay (3 μm pore size, Sigma, ECM510) was utilized to assess chemotaxis. Isolated murine lung fibrocytes were resuspended in DMEM with 0.1% bovine serum albumin (BSA, Sigma, A9418) to reach a density of 1 × 10⁶ cells/mL. Cell suspensions were placed in the upper chambers of the migration assay plate, while the lower chambers were filled with chemoattractants, such as lung-conditioned medium (LCM) or sphingosine-1-phosphate (S1P, Sigma, S9666) at concentrations of 10, 100, and 1000 nM, with or without FTY720 (Cayman Chemical, Ann Arbor, MI, USA; 10006292) at final concentrations of 1 μM or 5 μM. The plate was incubated overnight in a humidified environment containing 5% CO_2_ at 37 °C. Following incubation, any non-migrated cells in the upper chamber were carefully removed, and the migrated cells in the lower chamber were stained with CellTracker™ Green CMFDA dye (1:500, Thermo Fisher, C7025) for 30 min at 37 °C. The count of migrated fibrocytes was quantified by examining 10 high-power fields (HPFs) per well using a light microscope.

### 4.12. Flow Cytometry Analysis of Fibrocytes

Fresh lung tissues were finely minced with a scalpel and digested into single-cell suspensions using 0.2% collagenase I (Sigma, C0130) and 0.1% DNase I (Roche, Indianapolis, IN, USA; 11284932001) in DMEM for 2 h at 37 °C. The digested cells were filtered through a 70 μm cell strainer, washed, and then resuspended in PBS supplemented with 2% FCS (Gibco, 10099141). To prevent nonspecific binding, the cells were incubated with anti-mouse CD16/32 (1:100, Thermo Fisher Scientific, 14-0161-82) for 30 min at 4 °C. After washing, the cells were stained for 30 min at 4 °C in the dark with the following fluorophore-conjugated antibodies for 30 min: Anti-CD11b (Thermo Fisher, 25-0112-82, 1:200), Anti-Ly6G (Abcam, ab25377, 1:200), Anti-Gr-1 (Abcam, ab241738, 1:200), Anti-CD45 (Thermo Fisher, 48-0451-82, 1:200), and Anti-IL-5 (Abcam, ab253423, 1:600). Following two washes with PBS, the stained cells were resuspended in 500 μL of PBS containing 2% FCS. The percentage of CD45 and COL-I double-positive cells was detected using flow cytometry to evaluate the number of fibrocytes. Briefly, lung immune cells were suspended in 100 µL of PBS and incubated with FITC-conjugated CD45 and APC-conjugated CD11b (Thermo Fisher, USA) antibodies for 15 min at 4 °C. Afterward, cells were treated with Fix/Perm buffer and incubated with Biotin-conjugated Collagen I Polyclonal Antibody (Thermo Fisher, Rockland, MA, USA; 600-406-103) for 30 min in the dark, washed, and then stained with PE-conjugated Streptavidin. The percentage of fibrocytes was analyzed using a flow cytometer. 

### 4.13. RNA Extraction and Real-Time PCR

Total RNA was extracted from lung tissues utilizing the Qiagen RNeasy Mini Kit (Qiagen, Germantown, MD, USA, 74104) following the manufacturer’s guidelines. The concentration and purity of RNA were determined using a NanoDrop spectrophotometer (Thermo Fisher Scientific, ND-2000). cDNA was synthesized from 1 μg of total RNA with the SuperScript IV Reverse Transcriptase Kit (Thermo Fisher Scientific, 18091050) and random hexamer primers (Thermo Fisher Scientific, N8080127), adhering to the manufacturer’s instructions. Real-time PCR was conducted using the Applied Biosystems QuantStudio 5 System (Thermo Fisher Scientific) and PowerUp SYBR Green Master Mix (Thermo Fisher Scientific, A25742). Gene-specific primers were designed and employed according to standard protocols. GAPDH or β-actin was utilized as an internal control, and relative gene expression levels were calculated using the ΔCt method.

### 4.14. qPCR Assay Design and Validation

Primer information: (i) The forward and reverse sequences are listed in Supplementary Table S7; (ii) all primers were designed using NCBI Primer-BLAST, ensuring that they spanned exon–exon junctions and were evaluated in silico for potential secondary structures and off-target interactions. Specificity: Each assay produced a single peak in the post-run melt curve. Sensitivity and dynamic range: (i) Ten-fold serial dilutions (from 10^6^ to 10 template copies) of pooled lung-cDNA were analyzed in triplicate; (ii) the slopes of the standard curves ranged from −3.23 to −3.42, indicating amplification efficiencies of 96–104%; (iii) all assays demonstrated linearity across the complete five-log range, with R^2^ values ≥ 0.998; (iv) the limit of detection, defined as the lowest dilution yielding ≥95% replicate detection, was ≤10 copies for each gene. Precision: The intra-assay coefficient of variation was <1.5%, while the inter-assay CV was <2.5%.

### 4.15. Preparation of Conditioned Media from the Mouse Lungs

To generate the lung-conditioned medium, the lung tissue was thoroughly washed with fresh cold PBS until it was free of blood. The lungs were then placed in a 60 mm^2^ Petri dish for each mouse and minced into pieces approximately 1 mm^3^ in size using sterile scalpel blades. The tissue fragments were resuspended in an appropriate volume of DMEM/F-12 medium supplemented with antibiotics, and the tissues were incubated in a well of a 6-well plate per mouse at 37 °C with 5% CO_2_. After 24 h, the entire contents of each well were transferred into separate 50 milliliter conical tubes. The conditioned media were then diluted with equal volumes of fresh DMEM/F-12 medium for each tube. Large tissue debris was removed by centrifugation, and the conditioned media were pooled through a 0.22 micron syringe strainer into a single 50 milliliter conical tube. 

### 4.16. Statistical Analysis

All statistical analyses were performed using GraphPad Prism 9. Data are presented as the mean ± standard error of the mean (SEM) from independent biological replicates. Differences between groups were evaluated using two-tailed independent-sample *t*-tests. The normal distribution of the data was confirmed with the Shapiro–Wilk test, while Levene’s test assessed the homogeneity of variances; all datasets met these assumptions. A *p*-value of <0.05 was deemed statistically significant.

## 5. Conclusions

In conclusion, our study highlights the *SPHK1* signaling axis as a key regulator of fibrocyte-mediated pulmonary fibrosis. By employing integrative genetic and experimental methodologies, we demonstrate that targeting *SPHK1* or its downstream receptor, *S1PR1*, significantly reduces fibrocyte recruitment, collagen deposition, and fibrotic remodeling. These findings offer valuable mechanistic insights into the role of sphingolipid metabolism in IPF and suggest that *SPHK1* may serve as a promising therapeutic target for future intervention strategies.

## Data Availability

The datasets used and/or analyzed in the current study were all obtained through public databases with links included in the manuscript. If any further details about these datasets are needed, they can be provided upon reasonable request to the corresponding authors.

## Supplementary Materials

The following supporting information can be downloaded at: https://www.mdpi.com/article/10.3390/ph18060859/s1, Table S1: The data sources and information utilized in the study. Table S2: Sphingolipid metabolism pathway genes in the GeneCard database. Table S3: MR results for sphingolipid metabolism pathway genes and IPF. Table S4: PheWAS results for binary traits (SPHK1). Table S5: PheWAS results for continuous traits (SPHK1). Table S6: Mediation TSMR results for SPHK1 mediating plasma protein to IPF. Table S7: Real-time PCR primer sequences.

## Abbreviations

The following abbreviations are used in this manuscript:

IPF	Idiopathic pulmonary fibrosis
*S1p*	Sphingosine-1-phosphate
*S1PR1*	S1p receptor 1
*SPHK1*	Sphingosine kinase 1
MR	Mendelian Randomization
PheWAS	Phenome-wide association study
BLM	Bleomycin
PPI	Protein–protein interaction
LCM	Lung-conditioned medium
